# Blood pressure to height ratios as simple, sensitive and specific diagnostic tools for adolescent (pre)hypertension in Nigeria

**DOI:** 10.1186/1824-7288-37-30

**Published:** 2011-06-24

**Authors:** Chukwunonso ECC Ejike

**Affiliations:** 1 Department of Biochemistry, Michael Okpara University of Agriculture, PMB 7267 Umuahia, Abia State, Nigeria

**Keywords:** adolescents, blood pressure-to-height ratio, diagnosis, (pre)hypertension

## Abstract

**Background:**

The age-, gender-, and height-percentile requirements of the 'gold-standard' for the diagnosis of (pre)hypertension in adolescents make it time-consuming for clinicians and difficult-to-use by non-professionals. Simplified diagnostic tools are therefore needed. The use of blood pressure-to-height ratio (BPHR) - systolic BPHR (SBPHR) and diastolic BPHR (DBPHR) - has been reported in Han adolescents, but it requires validation in other racial groups. The diagnostic accuracy of SBPHR and DBPHR in a population of 1,173 Nigerian adolescents aged 11-17 years, was therefore studied.

**Methods:**

Blood pressures were measured using standard procedures and (pre)hypertension were defined according to international recommendations. ROC curve analyses were used to assess the diagnostic accuracy of BPHR in defining (pre)hypertension in this population. Sex-specific threshold values for SBPHR and DBPHR were determined, and thereafter used to define (pre)hypertension. The sensitivity/specificity of this method was determined.

**Results:**

The accuracies of SBPHR and DBPHR in diagnosing (pre)hypertension, in both sexes, was >92%. The optimal thresholds for diagnosing prehypertension were 0.72/0.46 in boys and 0.73/0.48 in girls; while for hypertension, they were 0.75/0.51 in boys and 0.77/0.50 in girls. The sensitivity and specificity of this method were >96%.

**Conclusions:**

The use of BPHR is valid, simple and accurate in this population. Race-specific thresholds are however needed.

## Background

The tracking of hypertension from adolescence (probably from childhood) into adulthood has been fairly established in the literature [1-3]. The prevalence of adolescent (pre)hypertension, even in developing countries, is rising steeply [4], probably due to urbanization [5] and the positive energy balance - typified by excess weight gain - that comes with it [6]. The presence of hypertension in adolescents may therefore lead to early manifestation of its sequelae, for instance, coronary artery disease [7]. This may increase mortality and definitely morbidity from these conditions - situations that are inimical to development in developing countries.

It is therefore imperative to detect (pre)hypertension early in adolescents since that would aid cardiovascular disease management in adult life. In fact, the US National High Blood Pressure Education Working Group on High Blood Pressure in Children and Adolescents recommends, in its fourth report, the initiation of blood pressure monitoring early in life [8]. However, the "gold standard" for the diagnosis of (pre)hypertension in adolescents is difficult to appreciate and use by parents and non medical professionals [because of the age-, gender-and height-specific standards for both systolic blood pressure (SBP) and diastolic blood pressure (DBP)] [7]. This has led to the search for alternative, less cumbersome diagnostic tools for adolescent high blood pressure. Moreover, racial differences in blood pressure patterns in adolescents stimulated the questioning of the validity of using one single diagnostic chart across different racial populations [9,10].

Recently, anthropometric variables, not limited to body mass index (BMI), have been shown to be predictive of hypertension in adolescents [6,11], and Lui *et al*. [7] reported that blood pressure to height ratios were both feasible and accurate as diagnostic tool for hypertension in Han adolescents of China, and proposed optimal thresholds for SBP to height ratio (SBPHR) and DBP to height ratios (DBPHR) for the said population. This study recognizes the racial differences in adolescent hypertension [9,10], and therefore evaluated the sensitivity and specificity of the Lu *et al*. [7] tool in detecting (pre)hypertension in Nigerian adolescents; and determined optimal thresholds for both SBPHR and DBPHR in the Nigerian population.

## Subjects and Methods

### Subjects

Two data sets were analyzed for the present study. The first was from a study of adolescent blood pressure and nutrition conducted in Kogi State of Nigeria [4,5,12]. The second was from a similar complementary study conducted in Umuahia, Abia State of Nigeria. The same protocol was used for both studies. Data from apparently healthy school children aged 11-17 years, numbering 1,173 (50.1% girls) from both locations were included for the present analysis. Only subjects who gave an informed verbal consent after consulting with their parents or legal guardians were allowed to participate in the studies. Adolescents who had overt signs of ill-health on physical examination or who admitted being on medications for any diseases were excluded from the study. No honoraria were paid to participants.

### Measurements

The ages of the subjects were obtained from their school records, and age at last birthday was recorded per subject. Height (for each subject) was measured, to the nearest 0.5 cm, using a non-elastic measuring tape, fastened to a vertical wall. The student was required to stand on bare feet during the measurement. For the measurement of weight, the students had to stand on bare feet and dressed in light clothing. An electronic weighing balance was used to measure each subjects' weight, to the nearest 0.1 kg. Using values from the height and weight measurements, Body Mass Index (BMI) was calculated using the formula BMI = Weight (kg)/[Height (m)]^2^. All the equipments were calibrated each morning according to the manufacturer's instructions.

The subjects were asked to rest for an initial 10 minutes, in a seated position and in a quiet room before their blood pressures were measured. Three separate blood pressure (BP) readings were taken per subject, at two minutes intervals, using an automated digital monitor (Omron HEM-741 CINT). Appropriate cuff sizes were used for each subject. The manufacturers put the error of measurement of the device at ± 3 mmHg. The first blood pressure reading was discarded and the average of the last two readings was recorded for both SBP and DBP of each subject. The same trained personnel took all measurements. To obtain the blood pressure to height ratios, the following equations were used: SBPHR = SBP (mmHg)/height (cm); DBPHR = DBP (mmHg)/height (cm) [7].

### Definitions

Normal blood pressure is taken as systolic and diastolic blood pressure that is < 90^th ^percentile for gender, age and height. Prehypertension is taken as systolic and diastolic blood pressure ≥ 90^th ^percentile, but < 95^th ^percentile for gender, age and height or ≥ 120/80 mmHg. Hypertension is taken as systolic and diastolic blood pressure ≥ 95^th ^percentile for gender, age and height [8].

Ethical approval was obtained from the Boards of the Departments of Biochemistry, Kogi State University, Anyigba, and Michael Okpara University of Agriculture, Umudike, Abia State. Further approvals were sought and obtained from the principals of participating schools.

### Statistical analysis

Means and standard deviations were calculated and differences between means separated by One-Way ANOVA, followed by *post hoc *multiple comparison tests. Numerical variables are presented as means ± standard deviations. The Pearson's product moment correlation coefficients were calculated and used to assess the relationship between SBPHR and DBPHR (each) and the other relevant variables. A significant threshold of *P *< 0.05 was employed for all analyses.

The receiver-operating characteristic (ROC) analysis was used to show the discriminatory ability/diagnostic accuracy of SBPHR and DBPHR (separately) with respect to separating subjects into normotensive, prehypertensive and hypertensive phenotypes defined by the age-, gender-and height-specific reference standards as presented in the 2004 Working Group charts [8]. The ROC curves were plotted using measures of sensitivity (ie true positive rate) and specificity (ie true negative rate) for the various cutoff points. Usually, the area under the curve (AUC) is a measure of this discriminatory/diagnostic power of a test. An AUC value of 1.0 indicates a perfectly accurate test whereas an AUC value of 0.5 indicates that the test performs worse than chance. That is, as diagnostic accuracy improves, the value for the AUC approaches 1. A test is considered accurate if it has an AUC value of ≥ 0.85 [7,13]. Optimal cutoff points for both SBPHR and DBPHR were determined by checking the cutoff point (from a range of possible cutoff points) that had specificity and sensitivity values that yielded the maximum sums from the ROC curves. Using the determined cutoff points as diagnostic tools, normotension, prehypertension and hypertension were defined in the population, and the sensitivity and specificity of the thresholds were calculated.

All data analyses were done using the statistical software package SPSS for windows version 17.0 (SPSS Inc., Chicago IL).

## Results

The means of the measured and calculated variables were found to be statistically similar (p > 0.05) between data from the two locations (within the respective ages and sexes) so the data were pooled and treated as one.

The clinical characteristics of the subjects are presented in Table 1. The mean heights of the boys and girls increased with age, but that of the girls plateaued from age 15 years. The boys were significantly (<0.05) taller than the girls from age 15 to 17 years. The mean weights of both boys and girls increased steadily with age and differed significantly (p < 0.05) only at ages 16 and 17 years when the boys were heavier than the girls. BMI for both sexes also increased with increasing age. However, the girls had significantly (p < 0.05) higher BMI compared to the boys at ages 14, 15 and 17 years. SBP for both sexes increased with increasing age, and there was no significant difference (p > 0.05) between the mean SBP values of the boys compared to the boys at any given age. Unlike SBP, DBP was significantly (p < 0.05) higher in the girls from age 15 to 17 years. SBPHR and DBPHR both followed the patterns observed for SBP and DBP respectively.

**Table 1:** Clinical characteristics of the studied adolescent subjects

	Boys	Girls	p
**Height (m)**			
11	1.52 ± 0.07	1.48 ± 0.07	0.033
12	1.51 ± 0.09	1.51 ± 0.06	0.796
13	1.54 ± 0.07	1.54 ± 0.07	0.859
14	1.57 ± 0.08	1.56 ± 0.07	0.419
15	1.60 ± 0.07	1.58 ± 0.08	0.020
16	1.63 ± 0.07	1.58 ± 0.06	<0.001
17	1.65 ± 0.07	1.58 ± 0.08	<0.001
Total	1.59 ± 0.09	1.57 ± 0.08	<0.001
**Weight (kg)**			
11	42.9 ± 5.5	41.6 ± 7.3	0.541
12	42.8 ± 7.5	44.4 ± 8.3	0.374
13	45.7 ± 6.7	46.7 ± 7.5	0.430
14	47.4 ± 7.7	48.9 ± 6.2	0.128
15	52.1 ± 7.1	52.3 ± 7.4	0.852
16	55.4 ± 7.9	52.8 ± 7.1	0.006
17	58.2 ± 8.7	55.5 ± 6.7	0.014
Total	50.8 ± 9.1	50.7 ± 7.9	0.904
**BMI (kg/m^2^)**			
11	18.5 ± 2.1	18.9 ± 2.7	0.557
12	18.6 ± 2.3	19.5 ± 3.1	0.237
13	19.2 ± 2.4	19.6 ± 2.6	0.336
14	19.3 ± 2.5	20.2 ± 2.7	0.021
15	20.3 ± 2.4	21.0 ± 3.7	0.042
16	20.9 ± 3.0	21.0 ± 2.7	0.740
17	21.3 ± 3.0	22.3 ± 3.5	0.024
Total	20.0 ± 2.8	20.7 ± 3.2	0.001
**SBP (mmHg)**			
11	104 ± 11	101 ± 11	0.446
12	105 ± 13	101 ± 14	0.357
13	104 ± 12	105 ± 12	0.886
14	109 ± 15	107 ± 14	0.225
15	111 ± 13	111 ± 13	0.913
16	116 ± 16	114 ± 14	0.437
17	114 ± 18	113 ± 16	0.506
Total	110 ± 15	110 ± 14	0.434
**DBP (mmHg)**			
11	55 ± 11	58 ± 10	0.288
12	56 ± 12	58 ± 8	0.380
13	58 ± 8	58 ± 9	0.959
14	59 ± 10	59 ± 10	0.813
15	58 ± 11	62 ± 11	0.005
16	58 ± 11	63 ± 12	<0.001
17	58 ± 13	63 ± 12	0.008
Total	58 ± 11	61 ± 11	0.004
**SBPHR (mmHg/cm)**			
11	0.69 ± 0.07	0.69 ± 0.08	0.990
12	0.69 ± 0.08	0.67 ± 0.08	0.353
13	0.68 ± 0.07	0.68 ± 0.08	0.796
14	0.70 ± 0.09	0.69 ± 0.09	0.407
15	0.70 ± 0.09	0.71 ± 0.09	0.367
16	0.71 ± 0.08	0.72 ± 0.09	0.307
17	0.69 ± 0.10	0.71 ± 0.12	0.074
Total	0.69 ± 0.09	0.70 ± 0.09	0.166
**DBPHR (mmHg/cm)**			
11	0.36 0.08	0.39 0.07	0.101
12	0.37 0.07	0.38 0.05	0.348
13	0.37 0.05	0.37 0.06	0.942
14	0.38 0.06	0.38 0.07	0.618
15	0.36 0.07	0.39 0.07	0.001
16	0.35 0.07	0.40 0.08	<0.001
17	0.35 0.08	0.40 0.08	<0.001
Total	0.36 0.07	0.39 0.07	<0.001

The numerical distribution of the subjects by age and sex is as follows: 11 years, 55 subjects (32 boys, 23 girls); 12 years, 65 subjects (36 boys, 29 girls); 13 years, 156 subjects (87 boys, 69 girls); 14 years, 215 subjects (108 boys, 107 girls); 15 years, 275 subjects (120 boys, 155 girls); 16 years, 233 subjects (109 boys, 124 girls); 17 years, 174 subjects (93 boys, 81 girls); Total, 1,173 subjects (585 boys, 588 girls).

The correlation coefficients (Table 2) show that both SBPHR and DBPHR were not significantly (p < 0.05) correlated with age and weight. Both were positively and negatively correlated, significantly (p < 0.001) with BMI and height, respectively. SBPHR was positively and significantly (p < 0.05) correlated with SBP; and DBPHR, in like manner, was positively and significantly (p < 0.05) correlated with DBP. Both correlation coefficients were very strong (>0.9). Conversely, SBP and DBP were positively correlated, significantly (p < 0.01) with all the anthropometric variables studied.

**Table 2:** Correlations between blood pressure to height ratios and other relevant variables in the studied adolescent population

		Age	Height	Weight	BMI	SBP	DBP
**SBPHR**	r(p)	+0.052 (0.073)	-0.015 (<0.001)	+0.060 (0.040)	+0.199 (<0.001)	+0.916 (<0.001)	+0.468 (<0.001)
**DBPHR**	r(p)	+0.002 (0.939)	-0.183 (<0.001)	-0.013 (0.660)	+0.140 (<0.001)	+0.422 (<0.001)	+0.957 (<0.001)
**SBP**	r(p)	+0.260 (<0.001)	+0.249 (<0.001)	+0.275 (<0.001)	+0.142 (<0.001)	-	+0.498 (<0.001)
**DBP**	r(p)	+0.111 (<0.001)	+0.100 (0.001)	+0.139 (<0.001)	+0.099 (0.001)	+0.498 (<0.001)	-

The AUC values for the accuracy of both SBPHR and DBPHR in diagnosing both prehypertension and hypertension in both sexes ranged from 0.925 to 1.000 (Table 3). The accuracy was higher in diagnosing hypertension (than for prehypertension) in both boys and girls. Also, the accuracy of DBPHR in diagnosing diastolic (pre)hypertension was higher than that of SBPHR in diagnosing (pre)hypertension.

**Table 3:** Areas under the ROC curves of SBPHR and DBPHR for diagnosing (pre)hypertension defined by age, gender and height-specific references

		AUC	P	**95% C.I**.
**Prehypertension**				
**SBP**	Boys	0.944	<0.001	0.925 - 0.962
	Girls	0.925	<0.001	0.900 - 0.949
**DBP**	Boys	0.989	<0.001	0.981 - 0.997
	Girls	0.987	<0.001	0.979 - 0.996
**Hypertension**				
**SBP**	Boys	0.989	<0.001	0.979 - 0.999
	Girls	0.990	<0.001	0.982 - 0.998
**DBP**	Boys	1.000	0.001	1.000 - 1.000
	Girls	0.998	<0.001	0.996 - 1.000

The optimal thresholds for diagnosing systolic prehypertension were 0.72 in boys and 0.73 in girls; while values for diastolic prehypertension were 0.46 for boys and 0.48 for girls. The optimal thresholds for diagnosing systolic hypertension were 0.75 in boys and 0.77 in girls; while values for diastolic hypertension were 0.51 for boys and 0.50 for girls. The sensitivities and specificities in each case were >82% (Table 4).

**Table 4:** Optimal thresholds of SBPHR and DBPHR for diagnosing (pre)hypertension in the studied adolescent population

	SBP		DBP	
	Boys	Girls	Boys	Girls
**Prehypertension**				
**Threshold**	0.72	0.73	0.46	0.48
Sensitivity	0.933	0.901	1.000	1.000
Specificity	0.839	0.828	0.970	0.949
**Hypertension**				
**Threshold**	0.75	0.77	0.51	0.50
Sensitivity	0.982	0.986	1.000	1.000
Specificity	0.956	0.963	0.998	0.991

Table 5 shows that the specificities and sensitivities of the determined thresholds - 0.72/0.46 and 0.73/0.48 for prehypertension in boys and girls respectively; and 0.75/0.51 and 0.77/0.50 for hypertension in boys and girls respectively - were all greater than 96%.

**Table 5:** Sensitivities and specificities for the optimal thresholds of SBPHR/DBPHR for diagnosing (pre)hypertension in the studied adolescent population

	Prehypertension		Hypertension	
	Boys	Girls	Boys	Girls
**Threshold**	0.72/0.46	0.73/0.48	0.75/0.51	0.77/0.50
Sensitivity	0.991	0.969	0.986	0.992
Specificity	1.000	1.000	0.998	1.000

## Discussions

The principal goal in the search to develop a new diagnostic tool for adolescent blood pressure assessment should be finding a method that is 'simple, inexpensive, easy to use and acceptable to the subjects' [7]. This is important because part of the reasons for a poor implementation of blood pressure monitoring in adolescents and therefore a high rate of undiagnosed hypertension in pediatric populations is the cumbersome nature of the widely accepted method for adolescent blood pressure definition which takes age, gender and height percentiles into consideration [14]. This "gold standard" is clearly too complicated to be used for self-assessment and blood pressure monitoring by the adolescents or their parents/guardians [7] and may be too time-consuming for health care professionals working in developing countries where few health care professionals have to attend to a very large proportion of the population. Furthermore, Hansen *et al *[14] report that despite the availability of charts and electronic programs for normal and abnormal blood pressures, pediatric clinicians still may find it difficult to integrate such facilities into their routine work flows. Though blood pressure is known to be related to age, gender and height in adolescents [4,8,15], a diagnostic tool that factors them in while removing the noted encumbrances would be ideal for blood pressure monitoring especially in resource-poor settings.

The blood pressure to height ratios (SBPHR and DBPHR) both take the height of the adolescent subject into consideration. The finding of an insignificant correlation between both SBPHR and DBPHR on the one hand, and age on the other hand, despite the significant positive correlation between SBP, DBP and age is consistent with the report of Lu *et al *[7] and implies that the conversion nullified the effect of age on blood pressure. The replacement of SBP and DBP with SBPHR and DBPHR respectively, therefore takes care of the effects of age and height, such that once both SBPHR and DBPHR are determined for both sexes, the diagnosis of hypertension in adolescents is simplified. The very strong positive correlation found between SBP and SBPHR, and between DBP and DBPHR lends credence to this substitution and is in consonance with the earlier report [7]. The inverse correlation between both SBPHR, DBPHR and height implies that shorter subjects had relatively higher values while taller subjects had relatively smaller values, therefore ensuring that tall adolescents (who have normal weights) are not misclassified as hypertensives or short and heavy adolescents are not misclassified as normotensives. Such cases of misclassification had been observed when a single age-specific blood pressure value was used [16].

The area under the ROC curves found in this study show that SBPHR and DBPHR have robust discriminatory capacities with respect to diagnosing hypertension (in this population). Applying the determined thresholds of 0.72/0.46 and 0.73/0.48 in defining prehypertension in boys and girls respectively, and 0.75/0.51 and 0.77/0.50 in defining hypertension in boys and girls respectively, discriminated effectively between the blood pressure phenotypes with sensitivities and specificities exceeding 96%. Lu *et al *[7] had reported such high discriminatory power, but got threshold values that were higher, than those presented here, for both stages 1 and 2 hypertension, and stage 2 hypertension. The said authors studied adolescents aged 13 to 17 years as against this study that recruited those aged 11 to 17 years. Variations in age may not however be responsible for the observed disparity in the threshold values in both studies, as each study reported an insignificant relationship between SBPHR, DBPHR and age. The differences in sample size between this report and the Lu *et al *report [7] may not explain the wide differences observed, as the sample size for this report is large enough to have good statistical power. The variations could be as a result of differences in race/genetics [9,10] or differences in socioeconomic and cultural factors pervading the different environments [5,17]. It is however noteworthy that the SBPHR and DBPHR method works in different populations. The observed differences in threshold values may only suggest that race-specific reference values are required.

This study may be limited by some factors. First, the not-too-large sample size for this study may imply a debatably reduced statistical power of the entire analyses. It was necessitated by cultural practices/belief systems that make people feel that subjecting themselves to a "new" test may predispose them to the disease in question, which is pervasive in the studied populations, thereby affecting the number of participants especially when no honoraria was paid for lack of funding. The subjects were naïve to blood pressure measurement [4]. Though a larger population may yield different threshold values, the differences may not be significant. These results therefore call for a very much larger national (or even regional) study aimed at determining cut off values for SBPHR and DBPHR in Nigeria (or perhaps West Africa). Second, blood pressures were measured using an oscillometric device instead of the standard auscultation protocol. It is known that blood pressure values measured by oscillometric equipments often vary from those got from auscultation [18]. Oscillometric devices have been shown, however, to be valid for use in children and adolescents [19,20]. Such equipments also eliminate the observer bias/human error inherent in using the auscultation protocol in epidemiologic studies.

## Conclusions

The use of sex-specific SBPHR and DBPHR proposed by Lu *et al *[7] is valid, inexpensive, accurate and easy to use in the studied population. It reduces drastically the number of threshold values that are important in diagnosing hypertension in adolescents. It would therefore be easily appreciated by non-medical professionals, especially adolescents, and medical professionals alike. It may also eliminate the under-diagnosis of adolescent (pre)hypertension, and in turn, help in the early management of cases, and ultimately a reduction in the morbidity and mortality arising from its sequelae.

## Competing interests

The author declares that he has no competing interests.

## Authors' contributions

CECCE solely designed the study, analyzed and interpreted the data and wrote the manuscript.
